# Emergency physician use of tissue Doppler bedside echocardiography in detecting diastolic dysfunction: an exploratory study

**DOI:** 10.1186/s13089-018-0084-5

**Published:** 2018-01-25

**Authors:** Marina Del Rios, Joseph Colla, Pavitra Kotini-Shah, Joan Briller, Ben Gerber, Heather Prendergast

**Affiliations:** 1 Department of Emergency Medicine, University of Illinois at Chicago, Chicago, Illinois USA; 2 Department of Cardiology, University of Illinois at Chicago, Chicago, Illinois USA; 3 Department of Internal Medicine, University of Illinois at Chicago, Chicago, Illinois USA

## Abstract

**Introduction:**

This study evaluates the agreement between emergency physician (EP) assessment of diastolic dysfunction (DD) by a simplified approach using average peak mitral excursion velocity (*e*ʹ_A_) and an independent cardiologist’s diagnosis of DD by estimating left atrial (LA) pressure using American Society of Echocardiography (ASE) guidelines.

**Methods:**

This was a secondary analysis of 48 limited bedside echocardiograms (LBE) performed as a part of a research study of patients presenting to the Emergency Department (ED) with elevated blood pressure but without decompensated heart failure. EPs diagnosed DD based on *e*ʹ_A_ < 9 cm/s alone. A blinded board-certified cardiologist reviewed LBEs to estimate LA filling pressures following ASE guidelines. An unweighted kappa measure was calculated to determine agreement between EP and cardiologist.

**Results:**

Six LBEs were deemed indeterminate by the cardiologist and excluded from the analysis. Agreement was reached in 41 out of 48 cases (85.4%). The unweighted kappa coefficient was 0.74 (95% CI 0.57–0.92). EPs identified 18 out of 20 LBEs diagnosed with diastolic dysfunction by the cardiologist.

**Conclusion:**

There is a good agreement between (*e*ʹ_A_) by EP and cardiologist interpretation of LBEs. Future studies should investigate this simplified approach as a one-step method of screening for LV diastolic dysfunction in the ED.

## Introduction

Diastolic dysfunction (DD) is an alteration of relaxation, filling, and/or distensibility of the left ventricle [1]. DD can lead to diastolic heart failure and increases the risk of readmission rates and in-hospital mortality [2]. The increased prevalence of DD has led to growing interest in early detection in acute care settings [3, 4].

The American Society of Echocardiography (ASE) guidelines outline a detailed algorithm for the diagnosis of DD which includes (1) spectral pulsed wave Doppler of transmitral inflow; (2) pulsed wave Doppler profile of pulmonary venous flow; (3) mitral annulus downward velocity measurements (*e*ʹ) using tissue Doppler imaging (TDI) at the septum (*e*ʹ_S_) and lateral wall (*e*ʹ_L_); and (4) left atrial (LA) volumes [5]. Obtaining these multiple measurements may be time-consuming and difficult for the average EP.

Average peak mitral annulus velocity by TDI (*e*ʹ_A_ = [*e*ʹ_S_ + *e*ʹ_L_]/2) has been described as an acceptable single-step method for assessing LV relaxation, using *e*ʹ_A_ < 9 cm/s as a threshold [6–9]. TDI measurements can be obtained in 30 s with nearly 100% success rate, even with poor echocardiographic windows [10, 11]. This simplified approach may be more suitable for use by EPs with limited experience in echocardiography.

The purpose of this study was to ascertain inter-rater agreement in DD determination between *e*ʹ_A_ < 9 cm/s measured by EPs and cardiologist interpretation of LBEs following the ASE guidelines.

## Methods

### Study design

This was a secondary data set analysis of LBEs completed as part of a prospective, cross-sectional with longitudinal follow-up study (details provided elsewhere) of patients presenting to the emergency department (ED) with asymptomatic elevated blood pressure [12, 13].

### Study protocol and measurements

LBEs were performed based on research staff availability by EPs (two emergency ultrasound fellowship-trained faculties and one emergency ultrasound fellow) who had performed at least 100 LBEs through routine clinical care and who underwent training and demonstrated proficiency in diastology with a board-certified cardiologist. A sonosite M-Turbo ultrasound system equipped with a harmonic 4.0-MHz variable-frequency phased-array transducer was used to obtain images and measurements. Studies were digitally archived for cardiologist review.

EPs utilized electrocardiogram (EKG) rhythm strips to time diastole. EPs determined *e*ʹ_A_ by averaging *e*ʹ_S_ and *e*ʹ_L_ measurements. EPs considered an *e*ʹ_A_ < 9 cm/s as evidence of DD without adjustment for age or other risk factors. A board-certified cardiologist with an ASE level III echocardiography certification independently reviewed LBE images while blinded to EP interpretation. The cardiologist rated the images in accordance to the 2009 ASE guidelines [8] and upon reviewing digital recordings of the following: parasternal long view for determination of LV wall thickness, apical four-chamber view for estimation of LA size, *E* and *A* measurements, *e*ʹ_S_ and *e*ʹ_L_, *E*/*e*ʹ ratios to assess LA pressure, estimation of LA size, and the EKG rhythm strip (see Table 1 for comparison of data interpretation).

**Table 1:** Comparison of data utilized by emergency physician vs. cardiologist for determination of diastolic dysfunction

Interpretation by	Data collected	Interpretation
Emergency physician	TDI measurements mitral annulus (i.e., *e*ʹ_S_ and *e*ʹ_L_)	Average TDI velocities at mitral annulus (i.e., *e*ʹ_A_)
	Sinus EKG rhythm strip	Timing of early and late diastole
Cardiologist	Clip of PSL view	LV wall thickness estimation
	Clip of apical 4-chamber view	LA diameter
	Mitral valve inflow velocities measurements (i.e., *E* and *A*)	*E*/*A* ratio
		*E*/*e*ʹ to estimate LA pressure
	TDI measurements mitral annulus (i.e., *e*ʹ_S_ and *e*ʹ_L_)	*E*/*e*ʹ to estimate LA pressure
	Sinus EKG rhythm strip	Timing of early and late diastole

TDI, tissue Doppler imaging; PSL, parasternal long, *e*ʹ_s_, mitral annulus downward velocity at the septum; *e*ʹ_L,_ mitral annulus downward velocity at the lateral wall; *e*ʹ_A,_ average mitral annulus downward velocity measured ([*e*ʹ_S_ + *e*ʹ_L_]/2); *E*, peak mitral valve inflow velocity in early diastole; *A*, peak mitral valve inflow velocity in late diastole; LA, left atrium; LV, left ventricle

### Data analysis

EPs and cardiologist indicated DD present, DD absent, or indeterminate for each LBE study. A 3 × 3 contingency table provided a summary of agreement. Inter-rater reliability between EPs and the cardiologist was determined using an unweighted kappa with 95% confidence interval (CI) coefficient using Stata Release 15, StataCorp.

## Results

Forty-eight studies were submitted to the cardiologist for review. Cardiologist and EP agreement are summarized in Table 2. Agreement was reached in 41 out of 48 cases (85.4%). The unweighted kappa coefficient was 0.74 (95% CI 0.57–0.92).

**Table 2:** Agreement between emergency physicians and cardiologist interpretation

Emergency physicians	Cardiologist		
	DD present	DD not present	Indeterminate^a^
DD present	18	0	1
DD not present	2	22	4
Indeterminate^b^	0	0	1

^a^Studies were rated “indeterminate” by cardiology based on the following: fused *E* and *A* waves (1), studies that met some criteria but not others (4), and clips with insufficient number of cycles recorded (1)

^b^One study was rated “indeterminate” by emergency physicians due to extremely disparate *e*ʹ septal and lateral measurements

## Discussion

Diastolic dysfunction is prevalent and delays in diagnosis can lead to increased morbidity and mortality. EPs with focused training in diastology may identify diastolic dysfunction with high sensitivity compared to a cardiologist trained in echocardiography. Previous studies have demonstrated that EPs can identify DD with high sensitivity, but either did not include TDI as part of their assessment [14] or reported only moderate agreement with cardiologist interpretation [4]. One study showed that EPs who met minimum requirements for LBEs based on American College of Emergency Physicians guidelines demonstrated high inter-rater agreement in the assessment of DD using primarily TDI, but failed to compare EP to a cardiologist interpretation [15]. Our study addresses the limitations of previous evidence by demonstrating that by following a more simplified approach using *e*ʹ_A_ alone, EPs can identify DD with high level of agreement compared to a cardiologist following the ASE guidelines.

## Limitations

Our sample size and convenience sampling may have introduced selection bias thus preventing a definitive correlation between *e*ʹ_A_ and DD. EPs did not screen for regional wall motion abnormalities. Because wall motion abnormalities of the left ventricular basal segments can influence mitral annulus TDI diastolic velocities, this may have led to an overestimation of DD prevalence. Moreover, comparison was limited to cardiologist interpretation of LBE images, which may not be representative of typical exams obtained by a technician or specialist. A larger, multi-center study comparing EP assessment of *e*ʹ_A_ against performance of a comprehensive echocardiogram can help establish external validity.

## Conclusions

This study highlights a promising simplified approach for identifying DD by EPs. Relying on *e*ʹ_A_ alone achieved good agreement for determination of DD compared to LBE interpretation by cardiologist. Future studies should further investigate this simplified approach as a one-step method of screening for LV DD in the emergency department.
